# Assembly of Microtubule Tactoids Through Condensates of PRC1 Constructs

**DOI:** 10.3390/biom16050684

**Published:** 2026-05-05

**Authors:** Julia Bourdeau, Imara Davis, Elaysia Johnson, Leying Shi, Yiling Lan, Kelsey Moody, Aaron Wolfe, Leila Farhadi, Heidi Hehnly, Jennifer L. Ross

**Affiliations:** 1 Department of Physics, Syracuse University, Syracuse, NY 13244, USA; jfbourde@syr.edu (J.B.); ejacks15@syr.edu (E.J.); lshi@colgate.edu (L.S.); lfarhadi@syr.edu (L.F.); 2 Department of Biology, Syracuse University, Syracuse, NY 13244, USA; imdavis@syr.edu (I.D.); ylan03@syr.edu (Y.L.); hhehnly@syr.edu (H.H.); 3 Department of Physics, Colgate University, Hamilton, NY 13346, USA; 4 Ichor Life Sciences, Inc., 2561 US Route 11, LaFayette, NY 13084, USA; kmoody@ichorlifesciences.com (K.M.); awolfe@ichorlifesciences.com (A.W.)

**Keywords:** microtubules, PRC1, microtubule-associated proteins, biomolecular condensates, mitotic spindle, self-organization, in vitro reconstitution

## Abstract

The cytoskeleton fulfills many essential roles that are necessary for cell function. Perhaps the most important of these roles is the formation of the mitotic spindle. The mitotic spindle is a complex and dynamic structure made up mainly of microtubules and numerous microtubule-associated proteins (MAPs), but the mechanisms behind its creation and regulation are not fully characterized. Recent research has focused on the link between liquid–liquid phase separation (LLPS) of MAPs and these microtubule organizations. Here, we focus on the protein regulator of cytokinesis 1 (PRC1), a spindle midzone-associated anti-parallel microtubule crosslinker. PRC1 is known to undergo LLPS to form condensed droplets without the need for additional crowders. Using confocal fluorescence microscopy and different constructs of PRC1, we investigate the ability of PRC1’s structural domains to form droplets and hierarchically organize microtubule tactoids. We find that the removal of PRC1’s unstructured C-terminal tail does not inhibit its ability to form droplets or microtubule tactoids in vitro. Removing part of PRC1’s N-terminal rod domain inhibits but does not completely suppress droplet formation.

## 1. Introduction

The interior structure of cells is organized and rearranged to allow cells to adapt and react to their environment. The cytoskeleton is the essential physical component for intracellular organization [1,2,3,4]. For instance, microtubules need to be dynamic and adaptable so that different geometries, such as the bipolar mitotic spindle, can be assembled and disassembled during cell division [5,6,7,8,9]. Yet, the complexity of the mitotic spindle, with its numerous protein components, the changing nature over time, and the difference between cell types, has made this system an exciting and still incompletely characterized biological organization that continues to be studied in live cells. There have been attempts to recreate it with a minimal purified system in vitro [10,11,12,13]. Many studies have succeeded in recreating biologically relevant microtubule organizations through the use of bottom-up in vitro reconstitution experiments. Spindle-like and aster geometries can be created via self-assembling and self-organizing microtubules in these experiments with the aid of active and passive crosslinking MAPs and motors [12,14,15,16,17,18,19,20].

Recently, liquid–liquid phase separation (LLPS) has been proposed as a possible mechanism for how cells organize and compartmentalize different structures and functions [21,22,23,24,25]. This work has demonstrated the ability of certain MAPs to form condensates through LLPS, which can then promote microtubule nucleation [25,26,27,28,29,30,31,32]. It has been suggested that this may play a role in how certain MAPs interact with and organize microtubules. Here, we will focus on the protein regulator of cytokinesis 1 (PRC1), an MAP from the MAP65/PRC1/Ase1 family, which has been found to aid in the formation and regulation of the mitotic spindle midzone during metaphase to late anaphase as well as crosslink microtubules in telophase [33,34,35,36]. PRC1 is an anti-parallel crosslinker of microtubules [17,37] and has been shown to undergo LLPS in vitro [32]. Previous studies have demonstrated the ability of PRC1-family proteins to organize microtubules into tactoids, which are finite-length tapered microtubule bundles [32,38,39,40,41,42]. Condensates of MAP65 are able to hierarchically organize microtubules by first forming droplets in vitro, which then concentrate tubulin locally and function as nucleation centers for microtubules to form tactoids or asters [32]. Both shapes, which feature tapered bundles of anti-parallel microtubules, are reminiscent of the bipolar microtubule organization of the mitotic spindle.

**Figure 1:**
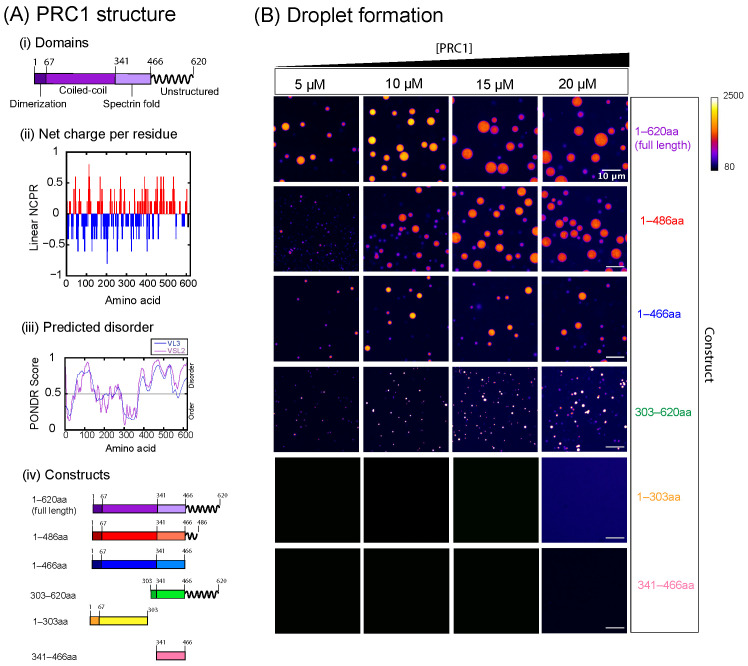
PRC1 constructs and condensation activity. (**A**) PRC1 structure and constructs. (**i**) Structural domains of full-length PRC1. (**ii**) Net charge per residue along the length of full-length PRC1 as predicted by local CIDER [44]. (**iii**) Disordered domains along the length of PRC1 as predicted by two different PONDR algorithms [45]. (**iv**) Constructs of PRC1 used in this study. (**B**) Fluorescence microscopy images of LLPS assays for each construct for 5 μM, 10 μM, 15 μM, and 20 μM protein concentrations. All images use the same fire look-up table with max 2500 and min 80 on a 16-bit scale. Scale bars for all images are 10 μm. Constructs 341–466 aa and 1–303 aa do not form droplets under these conditions.

For this study, we seek to investigate the same organizational phenomenon using purified human PRC1. We examine the contributions of PRC1’s structural domains to the formation of droplets and microtubule tactoids through the use of different constructs, each one omitting different parts of the protein. PRC1 is composed of an N-terminal rod-shaped domain that regulates its ability to form homodimers, a spectrin-like domain containing its microtubule binding site, and an unstructured C-terminal tail that also contributes to microtubule binding (Figure 1A) [37,43]. We find that removing the unstructured C-terminal tail from PRC1 does not inhibit its ability to form droplets, and that removing the majority of PRC1’s N-terminus results in more gel-like condensates. Only constructs that are capable of LLPS are seen to form microtubule tactoids, in agreement with previous results [39].

## 2. Materials and Methods

### 2.1. Protein Reagents

#### 2.1.1. PRC1

Human PRC1 WT full-length and construct plasmids are a gift from the Radhika Subramanian lab (Harvard Medical School). A detailed description of expression and purification protocols is provided in the supplementary materials of [37]. PRC1 is expressed using *Escherichia coli* BL21(DE3) Rosetta cells and purified using 6X His-tag affinity and passed through a size-exclusion column. Purified protein is aliquotted and stored at −80 °C in PRC1 storage buffer (50 mM sodium phosphate, pH 7.0, 150 mM NaCl, 5% glycerol and 30% sucrose). Constructs with GFP tags included full length, 1–486 aa, and 1–466 aa. Both labeled and unlabeled were made and combined at a 10% labeling ratio. Constructs 303–620 aa, 1–303 aa, and 341–466 aa were purified as unlabeled proteins. A subset was fluorescently labeled using Alexa488 dye (ThermoFisher A10235, Waltham, MA, USA) and mixed with the unlabeled to have a 10% labeling ratio.

#### 2.1.2. Tubulin

Lyophilized unlabeled and rhodamine-labeled tubulin from porcine brain is purchased from Cytoskeleton. Tubulin is resuspended to 5 mg/mL in PEM-80 buffer (80 mM PIPES, pH 6.8, 1 mM ${\mathrm{MgCl}}_{2}$, 1 mM EGTA) and centrifuged for 10 min at 110,000× *g*. Rhodamine-labeled tubulin is mixed with unlabeled tubulin for a final ratio of 10% fluorescently labeled. Tubulin is aliquotted and flash-frozen in liquid nitrogen and stored at −80 °C for single use.

### 2.2. In Vitro Assays

#### 2.2.1. LLPS Experiments

GFP or Alexa488-labeled PRC1 is combined at a 10% ratio with unlabeled PRC1 in PRC1 storage buffer and diluted to the desired concentration in PEM-20 buffer (20 mM PIPES, pH 6.8, 1 mM ${\mathrm{MgCl}}_{2}$, 1 mM EGTA). The protein is thawed and diluted on ice, then incubated for 25 min at 22 °C (RT). While incubating, a 20 μL flow chamber is assembled using a glass slide (Fisher) and silanized coverslip attached with two parallel strips of double-stick tape (3M). Silanization procedure can be found at [40]. A solution of 5% Pluronic F127 in water is pipetted into the chamber and left to incubate for 7–10 min at RT. After the sample is finished incubating for 25 min at RT, it is pipetted into the flow chamber using a small piece of filter paper to wick out the F127 as the protein sample is flowed through. The open ends of the chamber are sealed with wax and let to sit an additional 15 min upside-down at RT to allow droplets to settle to the bottom of the chamber via gravity, which improves imaging conditions.

#### 2.2.2. Microtubule Tactoid Experiments

For formation of microtubule tactoids, 13.6 μM fluorescently labeled tubulin and 1 μM PRC1 are combined in microtubule polymerization buffer PEM-20 with 0.25% 100 kDa polyethylene glycol, 1 mM GMPCPP (Jena biosciences), and an oxygen scavenging system composed of 25 mM DTT, 7.5 mg/mL glucose, 0.5 mg/mL glucose oxidase, and 0.15 mg/mL catalase. This mixture is pipetted into a flow chamber incubated with F127, and the ends are sealed with wax. The sample is then incubated for 30 min at 37 °C to allow for polymerization of microtubules. A detailed description for this procedure as performed using MAP65 instead of PRC1 can be found in [40].

### 2.3. Confocal Imaging and Photobleaching

Imaging of fluorescently labeled condensates and microtubule tactoids is performed using spinning disc confocal microscopy. All images are taken using an inverted Nikon Ti-E scope with a 100× oil immersion objective (NA 1.49), Yokagawa CSU-W1 spinning disk unit and Andor Zyla CMOS camera. Images are saved as .nd2 files (stacks of .tif files with metadata) using the Nikon Elements imaging software (v.5.21.03).

Photobleaching experiments are carried out using a 405 nm laser and Optomicroscan Laser FRAP module attached to the inverted Nikon Ti-E microscope. Timeseries data is taken as a confocal slice through the center of a droplet to observe the photobleaching and fluorescence recovery. A 1 μm diameter circular region of interest (ROI) in the center of the droplet is photobleached. For the construct 303–620 aa, droplets are too small to photobleach an internal circle, so the entire droplet is photobleached.

### 2.4. Droplet Image Analysis

#### 2.4.1. Droplet Diameter

For determining the size of droplets, images are taken as a confocal slice through their center and opened in FIJI/ImageJ (v.1.54s). The thresholding function is used to binarize the image so that droplets on the same focal plane can be separated from the background and stored as a list of ROIs using the Analyze Particles function in ImageJ. Once all droplets in the image are added to the ROI manager, droplet areas are exported to a data table and converted to diameters. KaleidaGraph (Synergy Software, Reading, PA, USA) is used to plot cumulative distribution functions and scatter plots and to compute statistics such as median values and standard error of the mean, which are represented as error bars on scatter plots.

#### 2.4.2. Partition Coefficient

Partition coefficient measurements are obtained by dividing the mean 16-bit intensity of each droplet in an image by the mean intensity of the dilute phase in that image. Droplets are detected using the same method as described in Section 2.4.1, and the intensity value for each droplet is exported to a data table. To obtain an average intensity measurement for the dilute phase, the threshold is adjusted in FIJI/ImageJ to exclude all droplets, including any fluorescence from droplets in other focal planes.

#### 2.4.3. Number Density

Median number densities are calculated by counting the number of droplets in each individual image frame (17,720 μm^2^ area) and calculating the median for each experiment. Droplets are detected using the same method as described in Section 2.4.1.

#### 2.4.4. Fluorescence Recovery After Photobleaching

Images of droplets and tactoids undergoing FRAP are characterized by opening timeseries data in FIJI/ImageJ. A circular ROI is drawn over the photobleached region and its intensity is measured over time. Intensity data is first corrected for background fluorescence as described in [46]. Data is then corrected for global photofading by measuring the intensity over time of a non-photobleached reference droplet in the vicinity of the photobleached droplet, as described in [47,48], and normalized according to the formula

(1) $$I_{norm}\left(t\right)=\frac{I_{corrected}\left(t\right)-min\left(I_{corrected}\right)}{I_{corrected}\left(0\right)-min\left(I_{corrected}\right)}$$

where $I_{norm}\left(t\right)$ is the normalized intensity over time, $I_{corrected}\left(t\right)$ is the intensity after correction for global photofading, $min(I_{corrected})$ is the minimum value of the corrected intensity, and $I_{corrected}\left(0\right)$ is the corrected intensity at t = 0 s [48]. Finally, the recovery portion of the curve is fit to equation

(2) $$F\left(t\right)=\frac{F_{0}+F_{\infty }\left(\frac{t}{t_{1/2}}\right)}{1+\frac{t}{t_{1/2}}}$$

found in [47,49] for fluorescence recovery of a circular photobleached region, where F(t) is the fluorescence intensity as a function of time, $F_{0}$ is the value of the fit at t = 0 s, $F_{\infty }$ is the asymptotic maximum of the fit as t goes to infinity, and $t_{1/2}$ is the characteristic halftime of the recovery. The mobile fraction of the photobleached region is defined as

(3) $$M_{f}=\frac{F_{\infty }-F_{0}}{F_{i}-F_{0}}\times 100$$

also as described in [47,49], where $M_{f}$ is the mobile fraction, $F_{\infty }$ is the fluorescence at t=∞, $F_{0}$ is the value of the fluorescence at time of bleach, and $F_{i}$ is the pre-bleach fluorescence. Due to the normalization of recovery curves, the mobile fraction is equivalent to the fitting parameter $F_{\infty }$ found in Equation (2). KaleidaGraph is used to perform calculations on data to correct it for global photofading, produce curve fits for fluorescence recovery curves, and produce all plots.

### 2.5. Tactoid Image Analysis

#### 2.5.1. Major and Minor Axis Length

Tactoids are detected using the same method in FIJI/ImageJ as used for droplet analysis. FIJI/ImageJ is used to draw and export the measurements for a best fit ellipse to each tactoid detected, including its angle, major axis length, and minor axis length.

#### 2.5.2. Tactoid FRAP

FRAP data for tactoids is analyzed using the same methods as described in Section 2.4.4 for both the 561 nm channel (tubulin) and 488 nm channel (PRC1) in order to compare fitting values between constructs.

## 3. Results and Discussion

### 3.1. PRC1 Is Capable of Phase Separation In Vitro Without Its Unstructured C-Terminal Tail

PRC1 is a mitotic-associated protein found in the nucleus until nuclear-envelope breakdown. It helps to organize the microtubules within the spindle midzone and the anti-parallel telophase bundles [33,34,36,50]. Full-length PRC1 consists of an N-terminal coiled-coil rod domain, a middle spectrin fold, and a Lys/Arg-rich unstructured C-terminal tail. The first 66 amino acids of the coiled-coil domain are responsible for PRC1’s homodimerization, the spectrin domain contains its microtubule binding site, and the disordered tail helps to augment binding affinity to microtubules (Figure 1A(i–iii)) [37,43]. We seek to identify which domains result in liquid–liquid phase separation of PRC1. We perform phase separation assays using five different constructs of purified fluorescently labeled PRC1. The first construct, 1–486 aa, omits one of two large basic residue clusters of the unstructured C-terminal tail [37], and the second construct, 1–466 aa, removes the entire unstructured tail. The third construct, 303–620 aa, omits the first 302 amino acids of the N-terminal rod domain, including the entirety of PRC1’s dimerization domain. Finally, 1–303 aa completely omits the spectrin domain and C-terminal tail, and 341–466 aa consists of the spectrin fold only (Figure 1A(iv)). For each experiment, PRC1 is diluted to the desired concentration in PEM-20 and left to incubate at room temperature in order to form droplets. The mixture is pipetted into a glass slide chamber for confocal imaging. We compare all the results to the full-length (1–620 aa) protein.

The constructs 1–303 aa and 341–466 aa are unable to undergo phase separation in our experiments in PEM-20 buffer at 22 °C (Figure 1B). All the other constructs formed observable droplets at 5 μM to 20 μM protein concentrations (Figure 1B). Full length, 1–486 aa and 1–466 aa display spherical droplets, whereas 303–620 aa shows condensates that are asymmetrical or appear aggregated (Figure 1B). From these results, we conclude that the unstructured C-terminal tail is not needed for droplet formation in the 1–466 aa construct, although unstructured/intrinsically disordered regions are often a driving force of the multivalent protein–protein interactions that result in LLPS [23]. PONDR algorithms VL2 and VSL3 predict that the N-terminus of PRC1 could also contain a disordered region based on its amino acid sequence (Figure 1A(iii)), which could provide an explanation for this effect as well as the fact that 303–620 aa formed aggregated condensates.

### 3.2. Rod and Unstructured Domains Affect Phase Separation of PRC1 In Vitro

**Figure 2:**
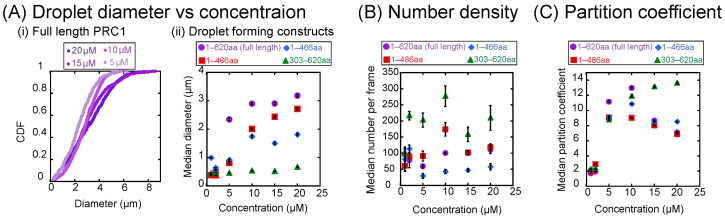
Quantification of droplet images taken by fluorescence microscopy. (**A**) Droplet diameters. (**i**) Cumulative distribution functions of droplet diameters plotted for full-length PRC1 at 5 μM (lightest filled circles), 10 μM (bright purple filled circles), 15 μM (medium purple filled circles), and 20 μM (darkest purple filled circles). (**ii**) Median droplet diameters as a function of concentration of PRC1 for full length (purple circles), 1–486 aa (red squares), 1–466 aa (blue diamonds), and 303–620 aa (green triangles). Error bars represent SEM. (**B**) Median number of droplets per image (17,720 μm^2^ area) of PRC1 for full length (purple circles), 1–486 aa (red squares), 1–466 aa (blue diamonds), and 303–620 aa (green triangles). Error bars represent SEM. (**C**) Median partition coefficients of PRC1 for full length (purple circles), 1–486 aa (red squares), 1–466 aa (blue diamonds), and 303–620 aa (green triangles). Error bars represent SEM. All data given in Table A1, Table A2, Table A3, Table A4 and Table A5.

Constructs that form droplets have increasing diameter with increasing PRC1 concentration, as seen in the raw images (Figure 1B) and verified by quantifying the droplet diameters (Figure 2A). This result is similar to what was observed for MAP65 [32]. The full-length construct consistently had the largest-diameter droplets compared to the other constructs (Figure 2A(ii)). Loss of the unstructured tail or rod domain results in smaller droplets, which we assume means a lower ability to phase separate (Figure 2A(ii)). Interestingly, PRC1 303–620 aa, which includes the entire C-terminal tail but is missing the majority of its N-terminal rod domain, makes droplets with the smallest median diameter at high concentrations. Thus, the ability of PRC1 to phase separate does not entirely depend on the intrinsically unstructured C-terminal tail region.

We can measure the density of droplets per frame (17,720 μm^2^ field of view) to further quantify the ability of the construct to phase separate (Figure 2B). Interestingly, the density of droplets per frame does not appear to show a dependence on concentration of PRC1; however, PRC1 303–620 aa consistently shows the highest number density of droplets for each concentration tested (Figure 2B).

We can also quantify the partition coefficient, a measure of how much protein is in the droplet compared to the background, using the intensity ratio inside and outside the droplet. We quantify a partition coefficient for each droplet by dividing the mean intensity value of the droplet by the mean intensity value of the background fluorescence in the same image. We find that the partition coefficient shows a non-monotonic trend for the full-length, 1–486 aa, and 1–466 aa constructs, but the trend is monotonically increasing for the 303–620 aa construct (Figure 2C). These trends are also visible in the images of the condensates (Figure 1B).

The decrease in the partition coefficient is a surprising trend that is different from what we observed with MAP65. As an explanation for this behavior, we hypothesize that the reduced partition coefficient at high concentrations is caused by a change in the ionic strength of the buffer since higher concentrations come with a higher amount of storage buffer. Next, we directly test the effect of buffer ionic strength on PRC1’s ability to form droplets.

### 3.3. Increasing Background Ionic Strength Hinders Phase Separation In Vitro

**Figure 3:**
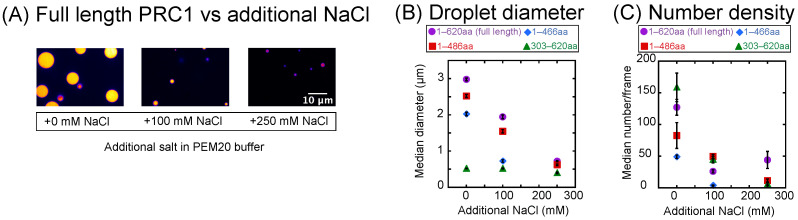
Effect of background ionic strength on droplets. (**A**) Example confocal images of droplets made from 15 μM full-length PRC1 in PEM-20 buffer with 0 added NaCl (**left**), 100 mM added NaCl (**middle**), and 250 mM added NaCl (**right**). Scale bar is 10 μm for all images. (**B**) Median droplet diameter with increasing background NaCl concentration for full length (purple circles), 1–486 aa (red squares), 1–466 aa (blue diamonds), and 303–620 aa (green triangles). Error bars represent SEM. (**C**) Median number of droplets per image for increasing background NaCl for full length (purple circles), 1–486 aa (red squares), 1–466 aa (blue diamonds), and 303–620 aa (green triangles). All data given in Table A6, Table A7, Table A8 and Table A9.

Since we suspect that the ionic strength of the buffer is affecting the droplet formation and partition coefficient, we investigate the effect of increasing salt concentration on droplet formation by performing experiments at a fixed protein concentration (15 μM). To this, we add additional NaCl to test the effects of increased ionic strength on the ability to form droplets. The condensation of all droplet-forming constructs is affected by adding additional NaCl, but the effects differ depending on the construct. For instance, full-length, 1–486 aa, and 1–466 aa constructs decrease their diameters as the added salt increases, but the 303–620 aa construct diameters are only minimally affected (Figure 3B). All the constructs show a decrease in the number of droplets formed (Figure 3C), with full-length PRC1 having a 65% decrease from 0 mM additional salt to 250 mM additional salt, 1–486 aa having an 86% decrease, and 303–620 aa having a 96% decrease. The 1–466 aa construct did not make any droplets at 250 mM additional NaCl. Our findings are similar to those of MAP65 droplets, which also show an inhibited ability to form droplets with increased background ionic concentration [32].

### 3.4. Mobility of PRC1 Molecules in a Droplet Depends on Construct and Age

**Figure 4:**
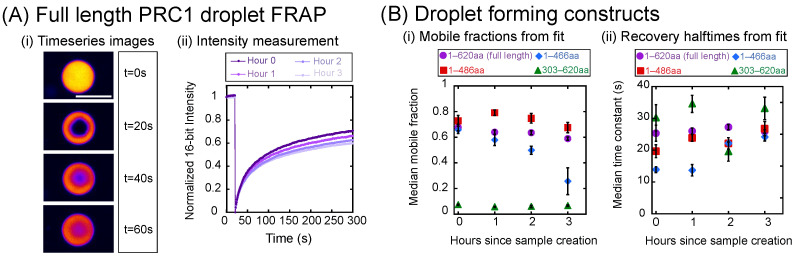
Quantification of mobility of molecules in condensates using FRAP. (**A**) Example FRAP experiment. (**i**) Timeseries of droplet before photobleaching (t = 0 s) at the first bleach (t = 20 s) and showing recovery over 60 s. Scale bar is 5 μm. (**ii**) Fluorescence recovery curves of droplets shown with increasing time since sample creation at 0 h (darkest purple), 1 h (medium purple), 2 h (lighter purple), and 3 h (lightest purple). (**B**) Comparison of mobility between constructs. (**i**) Median mobile fraction values for full length (purple circles), 1–486 aa (red squares), 1–466 aa (blue diamonds), and 303–620 aa (green triangles) calculated from fitting. (**ii**) Median recovery halftime values for full length (purple circles), 1–486 aa (red squares), 1–466 aa (blue diamonds), and 303–620 aa (green triangles) calculated from fitting. All data given in Table A10, Table A11, Table A12 and Table A13.

In order to compare the mobility of PRC1 molecules within droplets, we perform fluorescence recovery after photobleaching (FRAP) experiments. Droplets are formed for each construct at a concentration of 20 μM PRC1 in PEM-20, and a 1 μm circular region is photobleached to observe the resulting mobility of new fluorescent molecules into the region (Figure 4A(i)). Recovery curves are fit as described in Section 2.4.4 in order to extract values for the recovery halftimes and mobile fractions of each droplet measured (Figure 4A(ii)). Photobleaching experiments are performed at intervals of 1 h after sample creation for three hours in order to observe any effects of aging on the droplets (Figure 4A(ii), dark to light).

The mobile fraction is a measurement of the fraction of molecules that are able to diffuse in and out of the photobleached region. We find that the mobile fraction of the 303–620 aa construct is always low, but stable, over three hours of aging (Figure 4B(i)). These droplets, being asymmetric, are most likely to be gelated from early times. The FRAP results imply that no further “aging” or gelation of the condensates takes place. The molecules are already fairly immobile in the condensed phase. The other three constructs have a larger initial fraction of mobile molecules, with the full-length and 1–486 aa constructs being fairly stable in their mobile fraction over three hours (Figure 4B(i)). Despite starting off at hour zero with a similar mobile fraction of molecules to the full-length and 1–486 aa constructs, the 1–466 aa construct showed an approximately 60% decrease in this fraction over the course of three hours. Thus, this construct is the most prone to aging over time.

Recovery halftime is a measure of mobility and can be related to the diffusion coefficient within the droplet. Faster (shorter) times mean higher diffusion rates, and slower (longer) times mean slower diffusion. As expected, the gelated construct, 303–620 aa, typically has the longest recovery times, implying the slowest diffusion, which makes sense if the proteins are gelated (Figure 4B(ii)). The full-length and 1–486 aa constructs had intermediate recovery times and were similar to each other and slightly increased over three hours (Figure 4B(ii)). The 1–466 aa construct again demonstrated the most aging over time. The initial recovery times were the fastest, implying the highest diffusion coefficients, and, three hours later, the recovery times matched those of the full-length and 1–486 aa constructs (Figure 4B(ii)).

### 3.5. Condensates of PRC1 Can Organize Microtubules into Tactoid Shapes

**Figure 5:**
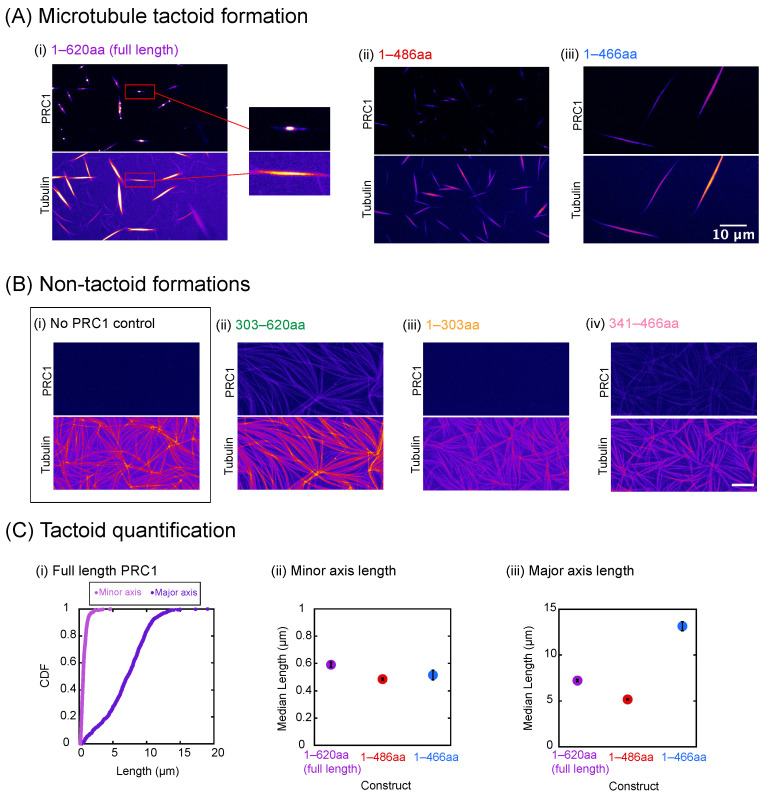
Tactoid formation by PRC1 constructs. (**A**) Fluorescent images of microtubule tactoids formed when (**i**) full length, (**ii**) 1–486 aa, and (**iii**) 1–466 aa PRC1 are incubated in the presence of tubulin and GMPcPP. Different channels for PRC1 (top) and tubulin (bottom) are shown. For (**i**), the boxed regions are zoomed in to show the colocalization of PRC1 and the tactoid. (**B**) Microtubule surface patterns formed with (**i**) no PRC1 present in the experiment, (**ii**) 303–620 aa, (**iii**) 1–303 aa, and (**iv**) 341–466 aa. Fluorescence channels for PRC1 (top) and tubulin (bottom) are shown. Scale bars for all images at 10 μm. (**C**) Quantification of tactoid images. (**i**) Cumulative distribution functions of tactoid minor axis lengths (light purple) and major axis lengths (dark purple) for full-length PRC1. (**ii**) Median lengths of tactoid minor axes for full length (purple circle), 1–486 aa (red circle), and 1–466 aa (blue circle). (**iii**) Median lengths of tactoid major axes for full length (purple), 1–486 aa (red), and 1–466 aa (blue). All data given in Table A14.

Previously, it was shown that MAP65, the plant analog of PRC1, could form microtubule tactoids, defined as thin, tapered, finite-length bundles, when microtubules were polymerized in the presence of the protein [32,38,39,40,41]. Further, the process to create these finite bundles is likely from nucleating microtubules from MAP65 droplets [32]. We seek to determine if PRC1 can also assemble microtubule tactoids from droplets of PRC1. In particular, we want to test the hypothesis that these microtubule organizations require the condensation of the droplet to control the nucleation and growth of the microtubules. For all six constructs, we polymerize microtubules in the presence of PRC1 protein, contained within a chamber formed by a glass microscope slide and coverslip. We find that the full-length, 1–486 aa, and 1–466 aa constructs are all able to form tactoids (Figure 5A) in these experiments, while the other constructs, including the gelating 303–620 aa construct, do not. However, the microtubules in these experiments form patterns similar to what is seen in the absence of an MAP65 or PRC1 crosslinker (Figure 5B).

For those that form tactoids, the pattern of the PRC1 on the microtubules is different depending on the construct. Specifically, a close-up view of a microtubule tactoid reveals that full-length PRC1 is highest in intensity and appears to stay more localized to the middle of the tactoid (Figure 5A(i)). The PRC1 pattern is more spread out for the 1–486 aa and 1–466 aa constructs compared to full length (Figure 5A(ii,iii)), binding more uniformly along the microtubules of the tactoid.

The other constructs are not able to induce microtubules to form tactoids. Constructs 303–620 aa and 341–466 aa each include PRC1’s spectrin domain, which contains its microtubule binding site, and are shown localizing to microtubule organizations. However, neither is able to make tactoids, likely due to them missing the first 66 amino acids of PRC1’s structure, which mediate its ability to dimerize. In the case of 1–303 aa, it does not appear colocalized to any microtubules due to the fact that it does not include any portion of the spectrin domain. As is consistent with our idea that droplets need to be formed in order for tactoids to grow, only the constructs of PRC1 that were capable of LLPS were also capable of forming tactoids. PRC1 303–620 aa, although it can form droplets, is unable to dimerize and therefore cannot crosslink microtubules into bundles.

For the PRC1 constructs that assist the formation of microtubule tactoids, we can measure the width (minor axis) and length (major axis) of the tactoids. For all the constructs, the widths have a very tight distribution around a half a micron (Figure 5C(i,ii)). This is the same as previously observed for MAP65 tactoids seen in several prior studies [38,39,41]. The tactoid length depends on the construct, with the median length of the 1–466 aa construct being the longest, the 1–486 aa construct the shortest, and the full length in between (Figure 5C(iii)). It is interesting that the 1–466 aa construct has the longest major axis since the unstructured tail helps to increase PRC1’s binding affinity to microtubules [37,43]. This structural difference appears to cause a change in tactoid formation.

A recent study of MAP65 tactoids found that increasing background ionic strength weakened MAP65 binding affinity to microtubules [41]. The organization of microtubules also transitioned from finite-length tactoids into long bundles, implying that the reduced affinity of MAP65 to microtubules causes tactoids to lengthen and eventually transition to bundles. Given that the binding affinity of PRC1 to microtubules is strengthened by its unstructured tail, it would appear that removing it entirely made the tactoids of PRC1 transition toward a longer, more bundle-like architecture.

### 3.6. PRC1’s C-Terminal Tail Mediates Its Mobility Within a Tactoid

**Figure 6:**
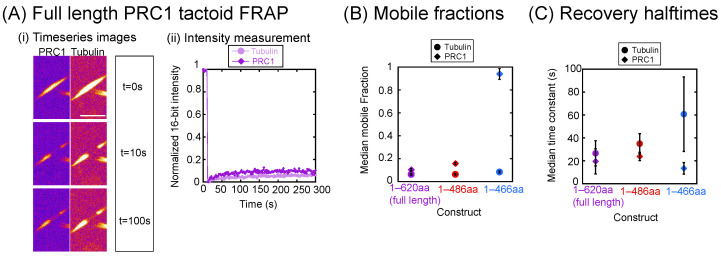
FRAP of PRC1 and tubulin in tactoids. (**A**) Example FRAP experiment data. (**i**) Timeseries of tactoid before photobleaching (t = 0 s) at the first bleach (t = 10 s) and showing recovery over 100 s for the PRC1 channel (left) and the tubulin channel (right). Scale bar is 5 μm. (**ii**) Fluorescence recovery curves for full-length PRC1 (dark purple) and tubulin (light purple). (**B**) Median mobile fraction values for full length (purple markers), 1–486 aa (red markers), and 1–466 aa (blue markers) for both tubulin (filled circle markers) and PRC1 (filled diamond markers). (**C**) Median recovery halftime values for full length (purple markers), 1–486 aa (red markers), and 1–466 aa (blue markers) for both tubulin (filled circle markers) and PRC1 (filled diamond markers). All data given in Table A15 and Table A16.

We perform two-color FRAP in order to observe the relative mobility of PRC1 and tubulin for the constructs that form tactoids (Figure 6A(i)). For each component, we quantify and fit the intensity recovery over time to determine the mobile fraction and recovery time, as we did for droplets. Since within tactoids we expect PRC1 to adopt a crosslinked conformation between microtubules, the mobile fraction represents the portion of PRC1 molecules that dissociate from the microtubules and are replaced by new fluorescent PRC1 (Figure 6B). For tubulin, which we expect to be highly constrained in the tactoid, the mobile fraction is only less than 10%. The PRC1 constructs are more mobile than tubulin, with the full length being the most immobile, the 1–486 aa construct being a bit more mobile, and the 1–466 aa construct being highly mobile and almost entirely able to recover. This is interesting because it has been previously reported that MAP65 is able to recover almost entirely [38]. We suggest that the recovery is a function of the binding constant for the PRC1–microtubule interaction, and it implies that the 1–466 aa construct has the weakest binding affinity of these three constructs. This is also consistent with the tactoid length data, which shows that this construct is likely the lowest-affinity variant, resulting in longer, more bundle-like organizations. All of this is consistent with prior works showing a modest microtubule-binding ability of the unstructured C-terminal tail [37].

For the tactoids, the recovery halftime data is likely to reveal information on the off rates for PRC1 binding to the microtubules in the tactoid (Figure 6C).This is because, when a molecule is bleached, the signal will remain dark until that molecule detaches and diffuses away. The replacement of the bleached molecule by a bright molecule should be fairly fast by comparison, so the off rate is likely the rate-limiting step and the step that we are measuring [51]. If that is the case, then the recovery time is measuring the inverse of the off rate. For the recovery halftimes, we again see that full length and 1–486 aa are similar, with values of 19.5 s and 23.7 s, respectively. However, the 1–466 aa construct is very different, taking almost half as long to recover as the longer constructs. This agrees with previous work that found PRC1 unbinding rates to tubulin are mediated by the unstructured C-terminal tail [37].

## 4. Conclusions

**Figure 7:**
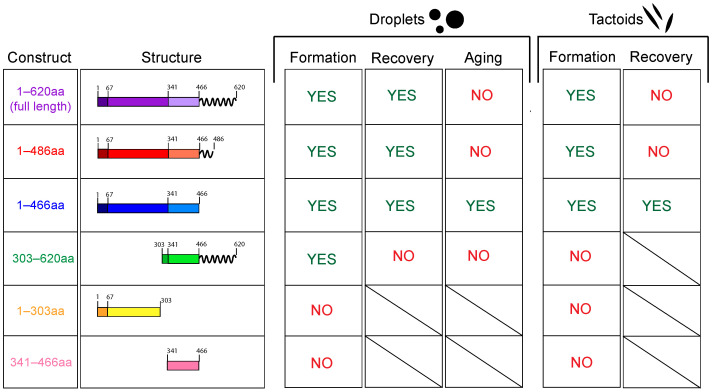
Summary of results with PRC1 constructs. Schematics of different PRC1 constructs used in experiments (**left**) and corresponding results in LLPS (**middle**) and tactoid (**right**) experiments. Blank fields indicate that this measurement is not applicable due to inability to form droplets or tactoids.

In this work, we have investigated the ability of PRC1 to undergo LLPS as a first step in the hierarchical organization of microtubules into tactoid shapes. Through the use of six different constructs of PRC1, we performed LLPS assays and microtubule polymerization assays in vitro in order to dissect which part of the molecule might be responsible for the formation of both condensed droplets and ultimately microtubule tactoid organizations. We observe that PRC1 is capable of phase separation either without the entirety of its unstructured C-terminal tail or without the majority of its N-terminal rod domain (Figure 7). However, it likely requires a combination of both of these domains to form the largest and most liquid-like droplets that are formed by the full-length protein.

In agreement with our hypothesis that PRC1 can hierarchically organize microtubules through phase separation, PRC1 constructs are only able to form tactoids if they are capable of phase separation (Figure 7). We also confirm that PRC1 needs to be able to dimerize and crosslink microtubules in order to form tactoids. The construct containing 303–620 aa, which can form misshapen droplets, is unable to form tactoids due to lacking the domain that mediates dimerization. In contrast, PRC1 1–466 aa is able to form droplets that are not misshapen but more prone to gelating over time. This construct can still form tactoids, although the binding affinity of PRC1 to microtubules is diminished compared to the full-length protein, resulting in a transition to a more bundle-like shape. Interestingly, this work also confirms that motor proteins are not necessary to obtain these spindle-like tactoid shapes. Passive crosslinkers, such as PRC1 in this case, are able to produce spindle-like organizations through the use of other properties, such as the ability to phase separate.

## Data Availability

The raw microscopy data supporting the conclusions of this article will be made available by the authors on request.

## Abbreviations

The following abbreviations are used in this manuscript:

MT	Microtubule
MAP	Microtubule-associated protein
PRC1	Protein regulator of cytokinesis 1
LLPS	Liquid–liquid phase separation
RT	Room temperature

## Appendix A. Droplet Quantification Values

**Table A1:** Median diameter values of droplets for scatter plot found in Figure 2A(ii). Uncertainty values are SEM.

	Median Droplet Diameter (μm)			
Conc. PRC1 (μM)	1–620 aa	1–486 aa	1–466 aa	303–620 aa
1	0.402 ±0.005	0.38 ±0.01	0.99 ±0.01	0.440 ±0.006
2	0.559 ±0.009	0.374 ±0.006	0.639 ±0.008	0.470 ±0.004
5	2.34 ±0.05	0.81 ±0.03	0.91 ±0.04	0.458 ±0.005
10	2.90 ±0.03	2.01 ±0.03	1.74 ±0.05	0.539 ±0.005
15	2.90 ±0.05	2.44 ±0.04	1.50 ±0.04	0.519 ±0.006
20	3.18 ±0.05	2.71 ±0.04	1.81 ±0.04	0.668 ±0.006

**Table A2:** Median number of droplets per image (17,720 μm^2^ area) for scatter plot found in Figure 2B. Uncertainty values are SEM.

	Median Number			
Conc. PRC1 (μM)	1–620 aa	1–486 aa	1–466 aa	303–620 aa
1	98 ±17	61 ±15	81 ±38	210 ±38
2	78 ±23	90 ±35	114 ±12	159 ±23
5	60 ±5	91 ±16	30 ±8	277 ±32
10	101 ±2	174 ±21	43 ±6	203 ±22
15	101 ±5	102 ±6	47 ±5	217 ±13
20	112 ±12	119 ±10	58 ±10	98 ±13

**Table A3:** Median partition coefficient values of droplets for scatter plot found in Figure 2C. Uncertainty values are SEM.

	Median Partition Coefficient			
Conc. PRC1 (μM)	1–620 aa	1–486 aa	1–466 aa	303–620 aa
1	1.707 ±0.006	1.96 ±0.02	2.26 ±0.02	2.24 ±0.03
2	1.92 ±0.01	2.76 ±0.01	2.20 ±0.03	2.24 ±0.03
5	11.2 ±0.1	9.09 ±0.08	9.2 ±0.1	8.8 ±0.1
10	13.0 ±0.1	9.04 ±0.05	10.85 ±0.08	11.9 ±0.1
15	8.67 ±0.05	8.04 ±0.04	8.51 ±0.08	13.1 ±0.1
20	7.11 ±0.06	6.88 ±0.04	8.53 ±0.06	13.6 ±0.1

**Table A4:** Number of droplets analyzed to compute median values found in Figure 2A(ii),C.

	Droplet *n*-Values for Diameter and PC			
Conc. PRC1 (μM)	1–620 aa	1–486 aa	1–466 aa	303–620 aa
1	991	601	1704	813
2	993	1135	1179	2122
5	496	1016	338	1814
10	999	1147	452	4325
15	979	1185	459	1798
20	1015	989	638	2884

**Table A5:** Number of images analyzed to compute median number droplets per image in Figure 2B.

	Image *n*-Values for Number Droplets Per Frame			
Conc. PRC1 (μM)	1–620 aa	1–486 aa	1–466 aa	303–620 aa
1	10	9	12	9
2	10	9	10	10
5	8	10	10	10
10	10	8	10	13
15	10	11	10	10
20	10	9	10	12

**Table A6:** Median diameter values of droplets vs additional NaCl for scatter plot found in Figure 3B. Uncertainty values are SEM. Construct 1–466 aa did not form any droplets at 250 mM additional NaCl.

	Median Droplet Diameter (μm) vs. Background NaCl			
Additional NaCl (mM)	1–620 aa	1–486 aa	1–466 aa	303–620 aa
0	2.98 ±0.04	2.52 ±0.04	2.02 ±0.02	0.519 ±0.006
100	1.94 ±0.05	1.55 ±0.05	0.73 ±0.03	0.52 ±0.01
250	0.72 ±0.01	0.62 ±0.02	N/A	0.40 ±0.02

**Table A7:** Median number of droplets per image vs additional NaCl for scatter plot found in Figure 3C. Uncertainty values are SEM.

	Median Number vs. Background NaCl			
Additional NaCl (mM)	1–620 aa	1–486 aa	1–466 aa	303–620 aa
0	127 ±13	83 ±20	49 ±3	159 ±23
100	26 ±3	50 ±4	4.0 ±0.3	45 ±5
250	44 ±14	12 ±3	0	6 ±2

**Table A8:** Droplet *n*-values for median diameters as a function of increasing NaCl in Figure 3B.

	Droplet *n*-Values for Diameter NaCl Scan			
Additional NaCl (mM)	1–620 aa	1–486 aa	1–466 aa	303–620 aa
0	2164	1113	1394	1798
100	419	439	42	536
250	1038	155	0	58

**Table A9:** Image *n*-values for median number of droplets per image as a function of increasing NaCl in Figure 3C.

	Image *n*-Values for Number Per Frame NaCl Scan			
Additional NaCl (mM)	1–620 aa	1–486 aa	1–466 aa	303–620 aa
0	17	10	29	10
100	15	10	11	12
250	17	10	4	8

**Table A10:** Median mobile fraction values from fitting for scatter plot found in Figure 4B(i). Uncertainty values are SEM.

	Median Droplet Mobile Fractions			
Hour	1–620 aa	1–486 aa	1–466 aa	303–620 aa
0	0.68 ±0.03	0.73 ±0.04	0.66 ±0.03	0.0731 ±0.0048
1	0.64 ±0.02	0.79 ±0.02	0.58 ±0.04	0.057 ±0.004
2	0.64 ±0.02	0.75 ±0.04	0.50 ±0.03	0.06 ±0.01
3	0.59 ±0.01	0.67 ±0.04	0.26 ±0.1	0.07 ±0.01

**Table A11:** Median recovery halftime values from fitting for scatter plot found in Figure 4B(ii). Uncertainty values are SEM.

	Median Droplet Recovery Halftime (s)			
Hour	1–620 aa	1–486 aa	1–466 aa	303–620 aa
0	25 ±3	20 ±2	14 ±1	30 ±4
1	26 ±1	24 ±1	14 ±2	35 ±3
2	23 ±1	22 ±2	22 ±2	20 ±3
3	25.9 ±0.5	27 ±2	24 ±1	33 ±4

**Table A12:** Number of droplets measured for each FRAP experiment/data point in Figure 4B(i,ii).

	*n*-Values for FRAP			
Hour	1–620 aa	1–486 aa	1–466 aa	303–620 aa
0	10	6	6	6
1	12	6	6	8
2	13	6	6	7
3	15	6	5	7

**Table A13:** *p*-values for distributions of droplet mobile fractions and recovery halftimes between hour 0 and hour 3. Represented by median values in Figure 4B(i,ii).

	*p*-Values Between Hours 0–3 Measurements	
Construct	Mobile Fraction	Recovery Halftime
1–620	6.1 × 10^−7^	0.12
1–486	0.93	0.14
1–466	0.18	0.0043
303–620	0.21	0.87

## Appendix B. Tactoid Quantification Values

**Table A14:** Median values for tactoid major and minor axis measurements from scatter plots in Figure 5C(ii,iii). Uncertainty values are SEM.

	Length (μm)	
Construct	Minor Axis	Major Axis
1–620 (n = 685)	0.59 ±0.02	7.2 ±0.1
1–486 (n = 647)	0.484 ±0.007	5.17 ±0.05
1–466 (n = 218)	0.51 ±0.04	13.1 ±0.5

**Table A15:** Median mobile fraction values for tactoid FRAP experiments from Figure 6B. Uncertainty values are SEM.

	Median Tactoid Mobile Fraction from Fitting	
Construct	Tubulin	PRC1
1–620 (n = 7)	0.063 ±0.008	0.10 ±0.01
1–486 (n = 9)	0.064 ±0.006	0.158 ±0.009
1–466 (n = 8)	0.084 ±0.009	0.94 ±0.05

**Table A16:** Median recovery halftime values for tactoid FRAP experiments from Figure 6C. Uncertainty values are SEM.

	Median Tactoid Recovery Halftime from Fitting	
Construct	Tubulin	PRC1
1–620 (n = 7)	27 ±11	20 ±11
1–486 (n = 9)	35 ±9	24 ±4
1–466 (n = 8)	61 ±33	13 ±5
